# Prescribing and using vitiligo treatments: lessons from a nested process evaluation within the HI‐Light vitiligo randomized controlled trial

**DOI:** 10.1111/ced.15193

**Published:** 2022-05-30

**Authors:** Paul Leighton, Joanne R. Chalmers, Jonathan M. Batchelor, Andy Rogers, Perways Akram, Rachel H. Haines, Garry D. Meakin, Jennifer White, Jane C. Ravenscroft, Tracey H. Sach, Miriam Santer, Maxine E. Whitton, Viktoria Eleftheriadou, Kim S. Thomas

**Affiliations:** ^1^ Centre of Evidence Based Dermatology, School of Medicine University of Nottingham Nottingham UK; ^2^ Department of Medical Physics and Clinical Engineering Nottingham University Hospitals NHS Trust Nottingham UK; ^3^ Nottingham Clinical Trials Unit University of Nottingham Nottingham UK; ^4^ Department of Paediatric Dermatology, Nottingham Children's Hospital Nottingham University Hospitals NHS Trust Nottingham UK; ^5^ Norwich Medical School University of East Anglia Norwich UK; ^6^ Primary Care and Population Sciences University of Southampton Southampton UK; ^7^ Walsall Manor Hospital Walsall UK

## Abstract

**Background:**

The HI‐Light Trial demonstrated that for active, limited vitiligo, combination treatment with potent topical corticosteroid (TCS) and handheld narrowband ultraviolet B offers a better treatment response than potent TCS alone. However, it is unclear how to implement these findings.

**Aim:**

We sought to answer three questions: (i) Can combination treatment be used safely and effectively by people with vitiligo?; (ii) Should combination treatment be made available as routine clinical care?; and (iii) Can combination treatment be integrated within current healthcare provision?

**Methods:**

This was a mixed‐methods process evaluation, including semi‐structured interviews with a purposive sample of trial participants, structured interviews with commissioners, and an online survey and focus groups with trial staff. Transcripts were coded by framework analysis, with thematic development by multiple researchers.

**Results:**

Participants found individual treatments easy to use, but the combination treatment was complicated and required nurse support. Both participants and site investigators felt that combination treatment should be made available, although commissioners were less certain. There was support for the development of services offering combination treatment, although this might not be prioritized above treatment for other conditions. A ‘mixed economy’ model was suggested, involving patients purchasing their own devices, although concerns regarding the safe use of treatments mean that training, monitoring and ongoing support are essential. The need for medical physics support may mean that a regional service is more practical.

**Conclusion:**

Combination treatment should be made available for people seeking treatment for vitiligo, but services require partnership with medical physics and ongoing training and support for patients.

## Graphical abstract

The HI‐Light Vitiligo Trial showed that hand‐held home‐based narrowband ultraviolet B phototherapy (NB‐UVB) in combination with potent topical corticosteroid (TCS) gives a better treatment response than potent TCS alone in people with active, limited vitiligo. Trial participants and healthcare professionals agreed that combination treatment with home‐based, hand‐held NB‐UVB and potent TCS should be made available to people with active, limited vitiligo. Some participants found it complicated to follow a regimen of combination treatment with TCS and hand‐held NB‐UVB. It was not always possible to predict which people were more likely to have difficulties. Concerns regarding the safe use of TCS and NB‐UVB mean that careful selection of suitable recipients for combination treatment, followed by adequate training, monitoring and ongoing support, are essential. Medical physics services need to be closely involved in the provision of home‐based hand‐held NB‐UVB treatment, to ensure that devices are properly checked and maintained. This may mean that regional, rather than local, provision is more practical

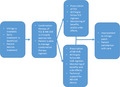

## Background

Vitiligo causes depigmented patches of skin and can have a considerable impact on quality of life. Two of the most commonly used vitiligo treatments are topical corticosteroid (TCS) and narrowband ultraviolet B phototherapy (NB‐UVB).^1^, ^2^, ^3^, ^4^ In the UK, people have mixed experiences of obtaining treatments for vitiligo, including TCS.^5^ NB‐UVB treatment is reserved for people with extensive vitiligo, and is administered using whole‐body cabinets in hospital settings. Provision of home‐based, handheld NB‐UVB devices is rare outside a small number of specialist centres.^6^, ^7^, ^8^, ^9^

The HI‐Light Vitiligo Trial^10^, ^11^, ^12^ was a three‐arm, double‐blind, randomized controlled trial (RCT) involving children (age ≥5 years) and adults with vitiligo limited to approximately 10% or less of the body and at least one active patch of vitiligo. Recruitment took place in 16 UK hospitals. Participants were randomized to receive potent TCS plus dummy NB‐UVB, handheld NB‐UVB plus dummy TCS, or a combination of potent TCS plus NB‐UVB. TCS (or dummy) was applied once daily on alternate weeks. NB‐UVB (or dummy) was used on alternate days, with dose adjustment if erythema occurred. Treatments were used for 9 months, with 3‐monthly clinic assessments, followed by 12 months of post‐treatment follow‐up to assess duration of treatment response.

The trial demonstrated that for people with active, limited vitiligo, combination treatment with potent TCS and home‐based, handheld NB‐UVB offers a better treatment response than potent TCS alone.^11^, ^12^ It also demonstrated that combination treatment offers better value for money than NB‐UVB or potent TCS used in isolation in the treatment of active, limited vitiligo.^13^

However, there are uncertainties in how best to implement this treatment combination in clinical practice. The benefits of this treatment combination (home‐based treatment, which reduces hospital visits; localized treatment, which minimizes exposure of unaffected skin to NB‐UVB) may need to be balanced against safety concerns, the complexities of combining treatment regimens, and the practicalities of testing and providing equipment within existing care pathways.

Here we report summary findings of a process evaluation nested within the HI‐Light Vitiligo Trial.^10^, ^11^, ^12^ Evaluating the opinions of stakeholders [people with vitiligo, parents of children with vitiligo, health service commissioners, healthcare professionals (HCPs)] we addressed three specific questions: (i) Can combination treatment be used safely and effectively by people with vitiligo?; (ii) Should combination treatment be made available as routine clinical care?; and (iii) Can combination treatment be integrated within current healthcare provision?

## Methods

This was a mixed‐methods process evaluation nested within the HI‐Light Vitiligo Trial and informed by the MRC guidelines for developing and evaluating complex interventions.^14^, ^15^ It included semi‐structured interviews with trial participants, structured interviews with commissioners and prescribers, an online survey of trial staff, and focus groups involving trial staff.

Full details of all aspects of the process evaluation are available in the funder's trial report.^12^

### Participants

A purposive sample of trial participants (including adults and young people, or their parents/carers) were approached for interview. Characteristics such as age, treatment group allocation, recruiting site and treatment success/failure (based on the primary outcome) were purposively sampled to achieve a maximum diversity sample (see Table 1).

**Table 1:** Interviewee characteristics: trial participants.

Group	*n*
Age group	
Parent of young person	10
12–17 years	2
≥ 18 years	13
Treatment group	
TCS	10
NB‐UVB	7
TCS + NB‐UVB	8
Treatment success^a^	
Yes	9
No	12
No primary outcome data	4

NB‐UVB, narrowband ultraviolet B; TCS, topical corticosteroid.

^a^ According to HI‐Light Trial primary outcome.

Dermatology service commissioners were identified via online directories of Clinical Commissioning Groups (CCGs) and via personal contact with members of the study team.

All site investigators (dermatologists and research nurses) from the 16 recruiting sites were invited to take part in an online survey and/or a focus group to review the delivery of combination treatment.

### Data collection

To avoid bias, the trial participants were approached to take part in an interview after they had completed the 9‐month treatment phase of the trial, and we included those with both ‘successful’ and ‘unsuccessful’ treatment, as judged by the primary outcome measure (Table 1). Interview questions considered treatment experience, the benefits and difficulties of combination treatment, and views about how combination treatment might be delivered/managed in the future.

Interviews with service commissioners considered topics such as awareness of vitiligo, local commissioning processes, and mechanisms that would support commissioning of new vitiligo treatments.

At the close of the study, all site investigators were emailed a link to an online survey (using Survey Monkey™ survey software). Survey questions covered potential challenges of delivering combination treatment and sought recommendations to support its future implementation. All questions included both fixed‐choice and free‐text response options. Site investigators were also invited to take part in an evaluation focus group to consider the implementation of combination therapy for vitiligo.

All interviews were conducted by telephone or video call while focus groups were face‐to‐face groups. All qualitative data were recorded using digital audio equipment.

### Data analysis

**Figure 1:**
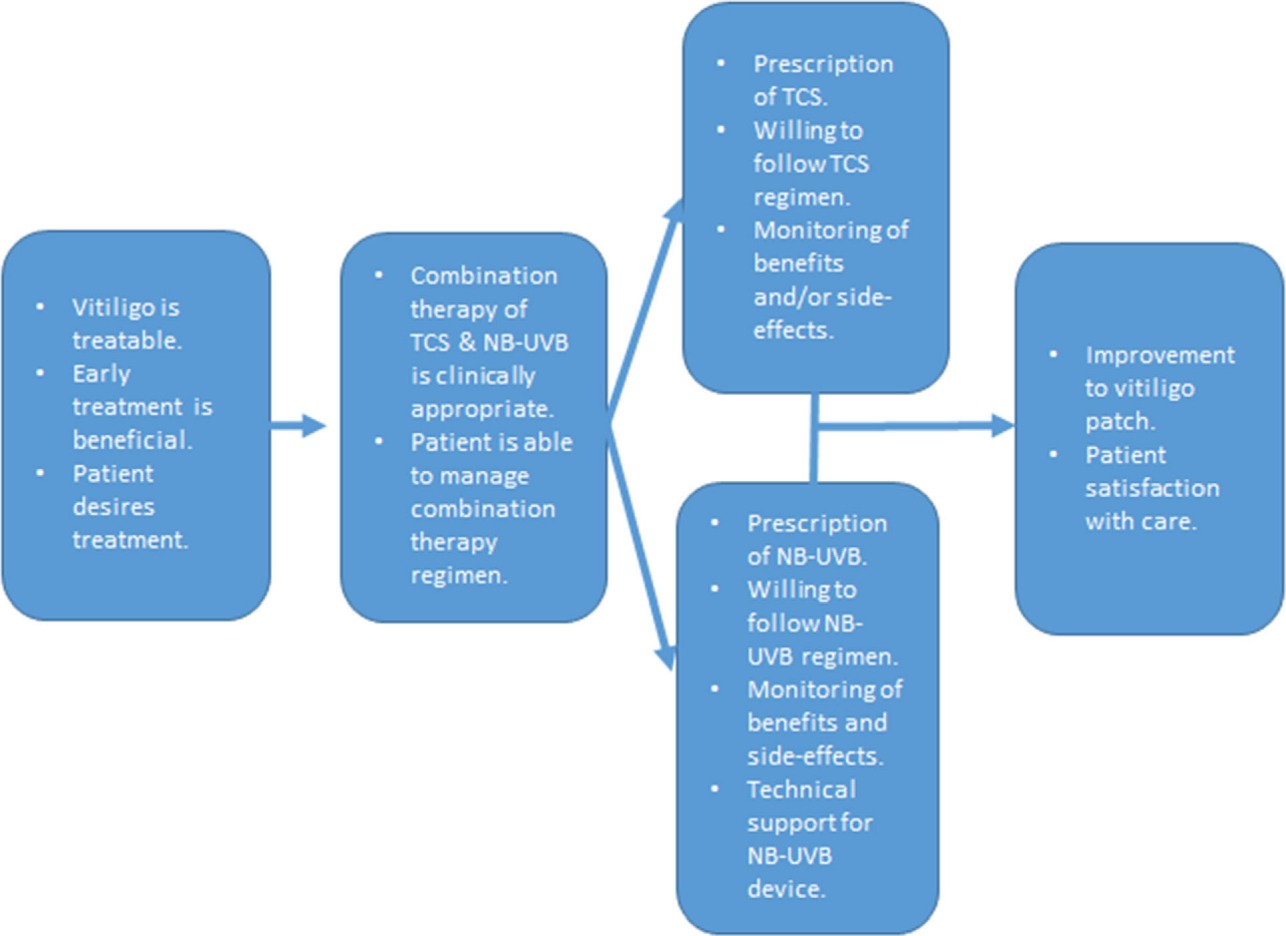
HI‐Light process evaluation logic model. NB‐UVB, narrowband ultraviolet B; TCS, topical corticosteroid. [Colour figure can be viewed at wileyonlinelibrary.com]

All recorded data were transcribed in full and handled using the NVivo software package^16^ (V12; QSR International, Burlington, MA, USA). Transcripts were coded following the conventions of framework analysis,^17^, ^18^ using a framework initially derived from an underpinning programme theory that described how combination therapy should be used^12^ (Fig. 1). Free‐text responses in the site investigator survey were mapped to this framework. The coding framework was developed and amended as data suggested new insight and topics. Coding and thematic development were checked independently by two team members (JC and PL) to ensure valid and relevant interpretation. Themes across matrices were compared, contrasted and synthesized to address the study objectives. Descriptive statistics were generated for the online survey responses.

## Results

We conducted 25 interviews with trial participants (Table 1), each lasting 30–60 min, and 9 shorter interviews (20–30 min) with service commissioners. In total, 25 site investigators (7 doctors, 16 nurses and 1 other) from the 16 recruiting sites responded to the online survey, and 13 (2 doctors and 11 nurses) participated in the focus group discussions.

The data presented here were responses to the three process evaluation questions.

### Question 1: Can combination treatment be used safely and effectively by people with vitiligo?

#### Using treatments individually

Most trial participants thought that the individual treatments were relatively simple and easy to use.

Some difficulties with light therapy were identified – such as parts of the body being difficult to reach, or devices breaking down or devices being damaged. The time commitment required for light treatment was a cause for comment, especially when treating multiple patches:

Adult participant 3: ‘*…it felt like an awful amount of time, I am pretty busy and to eventually be spending in excess of three quarters of an hour per two days just felt like an inordinate amount of time*.’
Adult participant 4: ‘…*[to start with] I was doing different parts of my body like six or seven [patches] or something … Then [because of the time] I just did three, the three patches they were interested in, so I was just treating them, no more*.’

By contrast, TCS was reported as easy to use, with the only issues being its greasy nature and poor absorption:

Adult participant 13: ‘*Yes, that's nothing; you just put it on before you went to sleep and you'd go to bed and I would just maybe be conscious of it for about twenty minutes to let it soak in and that was not a problem, the cream was not really an issue*.’

Interestingly, most of the trial participants did not raise concerns about adverse effects (AEs) of using a potent TCS on alternate weeks for up to 9 months.

None of these difficulties made either of the individual treatments unacceptable to participants.

#### Complexity in combining treatments

Although easy to manage individually, some site investigators were concerned that the complexity of managing two treatments in combination could be challenging for the people using them. Increasing or decreasing the NB‐UVB dose (as part of the treatment protocol or in response to erythema) seemed to confuse and cause difficulties for certain trial participants. Some site investigators were concerned that some individuals never fully understood the process of incremental dose change.

Site investigator focus group: Nurse 1 : *‘I think they struggled with the alternate days, I think they forgot about it, sometimes it didn't fit, they skipped a day if they had work commitments. It [the combination of treatments] complicated it terribly was the impression that I got…*'
Nurse 2: ‘*You can see that in their diaries, you can see the confusion, lots of crossings out …so confusing*.’

Data presented by trial participants suggested that this assessment was accurate for some:

Parent of child participant 5: ‘*Yeah I found it confusing for the first few weeks, it was like one week on one week off [for TCS], and every other day for the light and stuff*.’

#### The importance of support

Trial participants considered that the support of research nurses was essential in managing the treatment protocol (e.g. responding to erythema and assessing whether treatment was making a difference). They also recognized the value of a treatment diary to record treatment and AEs:

Adult participant 3: ‘*Yes, without that [treatment diary] it would be nowhere, without the form that you fill in with boxes I mean and writing down the time you would be absolutely nowhere, there's no chance in a million that you would actually keep to anything like the protocol*.’

Site investigators expressed concern about potential safety implications of the treatment:

Site investigator focus group: Nurse 7: ‘…*people do all sorts of things, they do*…'
Nurse 10: ‘…*you give them …something that it is relatively dangerous, UV light. We had patients who burnt their skin. I had a patient who decided he would try it out on the first patch he had years and years ago and did it for a random amount of time …a long duration …burnt his skin, a grade 4 burn …he didn't care*…’

Some trial participants acknowledged that they had misused accidentally (or willingly) the light treatment device:

Adult participant 3: ‘*I was completely knackered …at the end of the day, had done the light treatment. So, I sat and did my chest which was on the areas being treated and part of, one of my, part of my left hand which is the other bit of the treatment and then started to do the second bit on the left hand and fell asleep so I ended up burning myself*.’
Adult participant 11: ‘*I just ramped it up pretty much straight away back to what it was, but again no redness whatsoever which only really served to confirm it's a dummy*.’

### Question 2: Should combination treatment be made available as routine clinical care?

#### It should be made available

Although identified as potentially complex there was consensus that this type of combination treatment should be more widely available to people with vitiligo; 75% of site investigators (in the survey) agreed or strongly agreed with this. In the discussion, they reiterated this position, emphasizing that this is a clinical population with few treatment options.

Site investigator focus group: Nurse 9: ‘*We have always said that it [combination treatment] is the best of a bad bunch of treatments, and it probably still is. There is no fantastic treatment out there for vitiligo, there doesn't seem to be, and the trial doesn't show that it's fantastic. It's shown that for patients it's worthwhile doing because the quality of life is impaired for a lot of patients. They are pinning hopes on it*.’
Site investigator focus group: Nurse 1: ‘*I was really encouraged by the HI‐Light: results, that there was a positive*…’
Nurse 4: ‘*I think it's a disease with very limited treatments. And for that person living with that condition it has a massive impact …if combination treatment: was available that person would want to take it*.’

Commissioners reinforced that people with vitiligo have few treatment options available to them and that treatment pathways for vitiligo are often lacking.

Trial participants described a desire for access to treatment; in particular, parents of child participants were often quite desperate for any treatment that might offer hope of remission.

Parent of child participant 9: ‘…*we've been looking for a long time to find something like that because we've been at the NHS [National Health Service], and we were at a private doctor and nobody could not offer us anything except like some ointment, like cream and it was not really help …half of me was hoping that yes, something would work and it would help her, but if it didn't then we wasn't really going to lose anything*.’

These reflect the most common reasons motivating participation in the HI‐Light Trial. Some participants hoped that it would bring them access to new treatments for themselves or their children, while some subsequently hoped for complete remission and others hoped that their disease would stop spreading. For a minority of participants there was a sense of ‘nothing to lose’:

Adult participant 5: ‘…*had hoped it would totally recover the nine months or earlier you know, the sort of blemishes would disappear*.’
Adult participant 4: ‘*I decided to take part because why not, it would be working on my skin or not but I just decided to take part to see what happened*.’

#### It is not appropriate for all

However, neither the online survey respondents nor participants in the site investigator focus group indicated that combination treatment would be appropriate for all people.

Site investigators described how personal circumstances, such as mental health issues, other health problems or significant caring responsibilities (e.g. multiple children), might affect an individual's ability to follow a complex treatment regimen. They also said that people with unrealistic expectations of treatment response (e.g. rapid and dramatic improvements) might be less suitable candidates for combination treatment, as they might ignore the treatment regimen to accelerate improvements:

Site investigator focus group: Nurse 10: *‘It would be great if people did comply, and if it could be monitored. But, then not so great if people are not complying and using it as and when. That's my reservation*.’

Similarly, trial participants expressed the opinion that where improvement did not match expectations, an individual might prematurely cease treatment. Some of those trial participants allocated the dummy NB‐UVB phototherapy described their frustrations:

Adult participant 1: ‘*I think I only really found it onerous because I was just convinced it was a dummy, and I just felt as if I was …wasting [20 minutes] basically because I thought this was not going to be any good at all*.’
Adolescent participant 2: ‘…*as soon as I realised that it wasn't even tanning my skin I just, it was really hard to continue because it was really time consuming*.’

#### The ‘right’ candidate for combination treatment?

Site investigators concluded that it is difficult to predict which individuals will manage combination treatment well:

Site investigator focus group: Nurse 8: *‘yes, you can [choose the wrong patient for combination treatment] …some people who you think are going to be compliant, “yes they are grasping this really well.” Three months later they come back and you look at their diary and think “No!” they've been using the cream every day and the light for a week at a time … they've sort of switched it…‘*
Site investigator focus group: Nurse 1: ‘you don't really know [who will manage it well]…'
Nurse 2: *‘some people get it the first time, some the tenth time, some never get it*…'
Nurse 5: *‘I had a PhD level, a researchy person with vitiligo take part and her diary was as bad as any. It didn't really matter*…’

### Question 3: Can combination treatment be offered outside the research setting?

#### Is there a need for a new service?

Site investigators indicated that combination treatment for vitiligo might be delivered within broader dermatology phototherapy provision, and some indicated that they were already reusing devices in this type of setting for the treatment of vitiligo. The provision of medical physics services to maintain devices and specialist nurses to support home use was central to this.

However, commissioners considered it unlikely that dedicated services for vitiligo of this kind would be commissioned. They indicated that vitiligo might not be prioritized in commissioning discussions due to a perceived lack of demand from patients and HCPs for new services:

Commissioner 4: ‘*I'm not getting any complaints for example about the services that we provide. Like GPs aren't coming to me saying, we're not happy with this. As far as our GPs are concerned, they're getting a good service because their patients aren't complaining to them. It's not coming up on our monitoring in terms of performance*.'

Commissioners also indicated that vitiligo might be dismissed as a cosmetic (rather than clinical) problem:

Commissioner 2: ‘…*it could fall under cosmetic if it was on an area other than hands and face, which means that this wouldn't necessarily be a priority*.’
Commissioner 7: ‘*You do have a cosmetic exclusion policy. And that …that presumably will catch vitiligo within it*.’
Commissioner 3: ‘…*some people see it is as just a cosmetic problem*.’

#### Purchasing phototherapy devices privately

Site investigators recognized that handheld NB‐UVB devices can be bought online, and that positive findings in the HI‐Light Trial might encourage this. Most were uneasy about this and only 2 survey responders (out of 24) indicated that National Health Service (NHS) support for home‐based phototherapy was not important. Several trial participants described being tempted to purchase a NB‐UVB device, but expressed anxieties about ‘going it alone’:

Adult participant 6: ‘*I think they're about £100 aren't they? They're not fantastically expensive but I didn't then think I might go and buy one of those, largely because I wasn't sure how I would use it you know. It's very secure and comforting isn't it to have that kind of regime and do this, that and the other every day, and then you think “right okay so I know where I'm up to” and so on. So to suddenly be cut loose from that would be a little bit more you know, anxiety provoking, when you know that it's potentially dangerous*.’

#### Could there be a ‘mixed economy’ solution?

The potential for some form of ‘mixed economy’, where patients hire or purchase a NB‐UVB device within an NHS service, was mentioned in the focus group discussions as a way of reducing the economic burden on the NHS. Regarding this, site investigators stressed the importance of careful monitoring to ensure safe use of treatments, with an early follow‐up important to establish appropriate use and clinical benefit:

Site investigator focus group: Doctor 2: ‘…*we would have to spend a lot of time devising training programmes and making sure that everything is supervised … it would take a lot of investment to get everything up and running properly …to make sure that it is safe as well*.’
Parent of child participant 3: ‘*I personally think it needs an interim visit [before 3 months], if only to compare the photograph, because I do think that you forget what it was like and you do think “oh it's not making any difference,” but then when you see the photograph and you see the shape changing*…’

Several potential difficulties with a ‘mixed economy’ approach were flagged. Both trial participants and site investigators were concerned about unequal access for patients who cannot afford to purchase or hire a device. Some site investigators suggested that ‘purchasing healthcare’ might lead to unreasonable expectations and/or incorrect use, and that failure to return borrowed devices might challenge the viability of an NHS‐led service.

## Discussion

The HI‐Light Vitiligo Trial has demonstrated that 62% of participants receiving combination treatment gained some degree of benefit, with 27% achieving treatment success and 35% achieving partial treatment success.^11^ Site investigators were encouraged and felt that the results supported further implementation; however, comments from some commissioners suggest that the results may be insufficient to support new treatment pathways, with some commissioners considering vitiligo to be a cosmetic problem, even though research has shown this not to be the case.^19^ Despite these differences of opinion, interviews with trial participants and site investigators support the potential for home‐based, handheld, NB‐UVB phototherapy, as has been demonstrated previously,^8^, ^20^ and demonstrate the importance of offering new treatment options for people with vitiligo.

However, our evaluation also identified concerns about inappropriate use of NB‐UVB and about potentially harmful AEs. Previous studies of home‐based phototherapy have indicated that recipients need to be carefully selected^9^ and willing to follow treatment guidelines.^6^ ‘Reliable’ people are those who understand the treatment risk and can follow instructions,^21^ although unconscious bias could lead to potentially suitable recipients being denied treatment; for instance, having other health issues, not understanding the treatment or being unwilling to be clinically monitored might suggest candidates who are ‘unsuitable’ for home‐based phototherapy.^22^ Our evaluation reinforces that candidate selection is complex, and that home‐based phototherapy will not be suitable for all.

Our evaluation also highlights that predicting behaviour is difficult, and that a programme of training, monitoring and ongoing support is essential in the delivery of combination treatment for vitiligo. The importance of supervision^6^ and of regular follow‐up appointments to monitor treatment response^23^ have been recommended previously. Weekly phone contact and monthly outpatient visits have been proposed in a new NHS home‐based phototherapy service.^9^ Early and regular follow‐up contact may ensure appropriate use of TCS and NB‐UVB, limit side‐effects and help identify those struggling to manage the treatment regimen. Regular contact may also help HCPs to feel confident about the delivery of a home‐based treatment programme. A shared decision‐making tool has recently been developed,^24^ enabling people with vitiligo and HCPs to consider different treatment options, and to make joint decisions about which treatments might be most appropriate for a particular person, including whether they are likely to be able to use home‐based NB‐UVB safely and effectively.

Concerns around the safety of home‐based phototherapy support the involvement of medical physics departments in setting up and maintaining NB‐UVB devices. This is supported by our findings that the output of the NB‐UVB devices is quite variable, so they need to be checked thoroughly before use.^25^ This potentially limits the delivery of home‐based phototherapy and suggests that a regional, rather than local, service might be required (with medical physics services provided via a hub‐and‐spoke model).^6^, ^7^ This is in keeping with the comments from both site investigators and commissioners, who identified the economic constraints in creating novel, dedicated services for people with vitiligo. The NB‐UVB devices used in the HI‐Light Trial were purchased by the recruiting hospitals and remained their property on completion of the trial. However, despite the devices being available after the trial, very few sites had immediate plans to use the devices within pre‐existing phototherapy services. This was partly due to the costs and complexities of ensuring adequate medical physics oversight of the NB‐UVB devices and of providing adequate nursing input to ensure their safe use.

Internationally, the potential of handheld NB‐UVB devices for vitiligo treatment has been recognized,^26^, ^27^, ^28^, ^29^ and individual purchase or rental of phototherapy devices is common.^28^, ^29^, ^30^ There is some suggestion that private purchase and long‐term self‐management are linked with a greater incidence of AEs,^6^ reinforcing the importance of training and monitoring even where phototherapy devices are paid for personally. A Dutch service model, requiring individuals to demonstrate safe and appropriate use of a NB‐UVB device before it can be rented,^31^ seems a pragmatic solution to this. It is difficult to say whether or not a ‘mixed economy’ model for providing NB‐UVB devices would the best option within the NHS; the legal and logistical aspects of leasing devices may prove more complicated than providing the devices and training within pre‐existing phototherapy service frameworks.

In the process of running the HI‐Light Trial, we developed various resources that can be used to support delivery of a home‐based phototherapy service using handheld devices, including a training video, a dosing schedule and treatment diary/handbook, and instructions on how to measure minimal erythema dose. These are accessible via our website.^32^

The strengths of the study were that it was a comprehensive process evaluation, drawing upon the experiences of participants who received TCS plus NB‐UVB combination treatment for vitiligo, those who delivered the treatment and those who might commission it in the future. The findings complement the clinical and economic assessments reported elsewhere,^11^, ^13^ and provide an important context to inform future service development and delivery. Regarding limitations, we acknowledge that centres and participants were to some extent self‐selecting and that qualitative data analysis is in part an interpretative (rather than objective) process. Although we tried to avoid bias by including trial participants with both ‘successful’ and ‘unsuccessful’ treatment outcomes, all had continued treatment for the full 9‐month period and so their views may not have been fully representative. Further research with other patients, HCPs or a larger sample of commissioners may have yielded different findings.

## Conclusion

Although the combination treatment of TCS plus NB‐UVB is relatively complex to manage and will not be suitable for all patients, we found that, in the absence of other treatment options, people with vitiligo and HCPs were positive about its potential. Given the economic challenges of commissioning new services, a ‘mixed economy’ model of provision (where people with vitiligo purchase or hire devices) may be worth considering, although this would need to be explored in more detail, by directly asking people with vitiligo about their likely willingness to pay in such a model. Regardless of how the NB‐UVB devices might be provided, concerns regarding the safe use of TCS and NB‐UVB mean that training, monitoring and ongoing support to those using combination treatment are essential. The need for medical physics support may mean that a regional service is more practical than a local one.

**What's already known about this topic?**

- In the UK, people with vitiligo have mixed experiences of accessing treatment.
- NB‐UVB is used quite widely, but usually for extensive vitiligo, using whole‐body cabins in a hospital setting.
- The HI‐Light Vitiligo Trial showed that handheld home‐based NB‐UVB in combination with potent TCS resulted in better treatment responses than potent TCS alone in people with active, limited vitiligo.

**What does this study add?**

- Both trial participants and HCPs agreed that combination treatment with home‐based, handheld NB‐UVB and potent TCS should be made available to people with active, limited vitiligo.
- Some participants found it complicated to follow a regimen of combination treatment with TCS and handheld NB‐UVB, and it was not always possible to predict which people were more likely to have difficulties.
- A perceived lack of demand for treatment, or views that vitiligo is mainly a cosmetic problem, may be potential barriers to the commissioning of new services providing home‐based handheld NB‐UVB for vitiligo.
- Concerns about the safe use of TCS and NB‐UVB mean that adequate training, monitoring and ongoing support are essential
- Medical physics services need to be closely involved in the provision of home‐based handheld NB‐UVB treatment, to ensure that devices are properly checked and maintained, which may mean that regional, rather than local, provision is more practical

## Conflict of interest

All authors' organizations received financial support from the trial funder; none of the authors received any additional support from any organization for the submitted work. The authors declare that there are no other conflicts of interest.

## Funding

This study was funded by the National Institute for Health Research (NIHR) Health Technology Assessment Programme (project reference 12/24/02). The views expressed are those of the authors and not necessarily those of the NIHR or the Department of Health and Social Care. Support for this trial was provided through Nottingham Clinical Trials Unit, the UK Dermatology Clinical Trials Network and the NIHR Clinical Research Network. This paper represents a summary of the results of a nested process evaluation within the HI‐Light Vitiligo Trial. A full and detailed trial report has been published within the NIHR Journal and copyright retained by the Crown.

## Ethics statement

The study was approved by NRES Committee East Midlands – Derby (reference 14/EM/1173, SA04), and registered on 8 January 2015 as a clinical trial (no. ISRCTN17160087). All trial participants provided written informed consent.

## Data availability

The data that support the findings of this study are available on request from the corresponding author. The data are not publicly available due to privacy or ethical restrictions.
